# The Causal Effect of Basal Metabolic Rate on Type 2 Diabetes: A Two-Sample Mendelian Randomization Study

**DOI:** 10.1155/jdr/6523642

**Published:** 2025-05-10

**Authors:** Yu Huang, Jianxuan Wen, Xiaofeng Liao, Guiling Chen, Xiang Zeng, Yu Chen

**Affiliations:** ^1^Laboratory of Stem Cell Clinical Research, The Second Affiliated Hospital of Guangzhou University of Chinese Medicine (Guangdong Provincial Hospital of Chinese Medicine), Guangzhou, China; ^2^The Department of Endocrinology, The Second Affiliated Hospital of Guangzhou University of Chinese Medicine (Guangdong Provincial Hospital of Chinese Medicine), Guangzhou, China; ^3^The Department of Acupuncture, The Second Affiliated Hospital of Guangzhou University of Chinese Medicine (Guangdong Provincial Hospital of Chinese Medicine), Guangzhou, China; ^4^State Key Laboratory of Dampness Syndrome of Chinese Medicine, The Second Affiliated Hospital of Guangzhou University of Chinese Medicine, Guangdong Provincial Hospital of Chinese Medicine, Guangzhou, China

**Keywords:** basal metabolic rate, genome-wide association study, Mendelian randomization, sensitivity analysis, Type 2 diabetes

## Abstract

**Aim:** This study is aimed at systematically investigating the potential causal impact of basal metabolic rate (BMR) on the risk of Type 2 diabetes (T2D).

**Methods:** Data pertaining to single-nucleotide polymorphisms (SNPs) associated with BMR and T2D were gathered through a genome-wide association study (GWAS). Employing T2D as the dependent variable and BMR as the independent variable, SNPs displaying significant correlation with BMR were identified as instrumental variables (IVs). We also performed multivariable MR (MVMR) analyses using two different BMR datasets. The connection between BMR and the risk of T2D was scrutinized using the inverse-variance weighted (IVW) method, and a sensitivity analysis was executed to evaluate heterogeneity and pleiotropy.

**Results:** A potential causal relationship between higher BMR and increased T2D risk was observed (odds ratio (OR), 1.49; 95% confidence interval (CI), 1.31–1.7; *p* < 0.001). Significant heterogeneity was identified (Cochran's *Q* test, *p* < 0.001). However, sensitivity analyses demonstrated the robustness of the findings, with no evidence of horizontal pleiotropy and consistent results in leave-one-out tests. The MR-PRESSO test identified no outliers, confirming the absence of unknown pleiotropic effects. MVMR analyses, however, showed that the evidence became weaker after conditioning on BMI.

**Conclusion:** Our study provides robust evidence of a causal link between higher BMR and increased T2D risk. Despite heterogeneity, sensitivity analyses support our findings, warranting further research to confirm results and explore underlying mechanisms.

## 1. Introduction

Diabetes mellitus (DM) is now acknowledged as a prevalent health issue, with a persistent rise in cases observed worldwide. Type 2 diabetes (T2D) is identified by chronically elevated blood glucose levels, marking it as a metabolic disease [1]. Diabetes, as it unfolds, leads to a range of systemic complications, affecting the blood vessels, heart, kidneys, eyes, and nerves [2]. The prevalence of T2D is persistently on the rise globally, driven by the increasing rates of obesity, high-calorie diets, sedentary lifestyle, and an aging population. China and India are experiencing a diabetes prevalence of 10%, signaling a significant health challenge in these nations [3]. The diagnosed cases of T2D in Europe reached an estimated 60 million in 2017 [4]. The onset of T2D is influenced by the intricate interplay of genetic, environmental, and metabolic factors [5]. Understanding the clinical characteristics of T2D and exploring potential risk factors become imperative as we navigate its complexities.

Described as the daily energy metabolic rate, basal metabolic rate (BMR) is necessary for individuals to sustain the integrity of essential functions in both awake and steady-state conditions [6]. Accounting for approximately 60%–70% of total daily energy expenditure in humans, BMR assumes a critical role in regulating energy balance [7]. Thus, it is of significance to clarify the linkage between BMR and the occurrence of T2D. Considered an independent risk factor for death, BMR has gained widespread attention in various diseases [8–10]. Evidence from prospective cohort studies has linked higher BMR to increased risk of developing T2D. A study in Pima Indians found that BMR was a significant predictor of T2D incidence over a 5-year period, even after adjusting for overall adiposity [11]. Other observational studies in populations including Caucasians [12], African American [13], and Chinese [14] have reported similar associations between elevated BMR and greater risk of future T2D.

Possible mechanisms proposed to explain these observational associations include insulin resistance induced by intrinsic mitochondrial uncoupling and metabolic inefficiency [15], chronic sympathetic overactivity driving gluconeogenesis and lipolysis [16], and compensatory overeating leading to weight gain and obesity [17]. However, the observational design of previous studies limited causal inference due to potential residual confounding and reverse causation.

Using genetic variants as instrumental variables (IVs), Mendelian randomization (MR) examines the causal link between an exposure—such as a lifestyle-related or environmental risk factor—and an outcome, like the risk of a disease [18]. Acting as unconfounded markers of exposure, these genetic variants are considered provided they do not exhibit an association with disease risk through an alternative mechanism [18]. Due to their random assignment at conception, these genetic variants are not influenced by reverse causation. Thus, MR stands as an approach orthogonal to traditional observational epidemiological studies. In the absence of violations to the assumptions of MR, the connection between the genetic instruments and the outcome implies a causal relationship between the exposure and outcome. The random assignment of variants at conception in MR draws parallels with randomized controlled trials (RCTs), as both approaches similarly navigate some of the limitations of observational epidemiological studies [19].

In this study, we performed a two-sample MR analysis to investigate the causal relationship between BMR and T2D risk. By integrating large-scale GWAS summary statistics, we examined the potential causal effect of genetically predicted BMR on T2D liability. This study provides novel insights into the genetic relationship between BMR and the risk of T2D by using MR to explore the causal effects of BMR on T2D. By leveraging genetic variants as IVs, our research helps to clarify the role of BMR in T2D development, filling an important gap in the understanding of energy metabolism mechanisms underlying T2D. This work contributes to the growing body of evidence linking BMR with diabetes risk, offering potential pathways for targeted interventions.

## 2. Materials and Methods

### 2.1. Study Design

Utilizing a two-sample MR design, the current study ensured the validity of potential causal effects by requiring MR analyses to meet three core assumptions [20]. (1) In order to study the relationship between BMR and T2D, we need to find those genetic variants that are known to be associated with BMR. For example, certain genetic polymorphisms may affect mitochondrial function or energy expenditure, thereby modulating an individual's BMR. These genetic variants should reliably predict BMR levels in individuals. (2) In studying the relationship between BMR and T2D, we assume that genetic variants are randomly assigned before birth and are not influenced by acquired factors such as environment and lifestyle. This means that these genetic variants are not interfered by factors such as dietary habits, physical activity, and socioeconomic status. (3) When studying the relationship between BMR and T2D, the selected genetic variants should only indirectly affect the risk of T2D by influencing BMR and not directly act on the mechanism of T2D. For example, a genetic variant associated with BMR should not simultaneously affect insulin sensitivity or *β*-cell function unless this effect is mediated by altering BMR. This study was conducted and reported following the Strengthening the Reporting of Observational Studies in Epidemiology Using Mendelian Randomization (STROBE-MR) guidelines [21]. The completed STROBE-MR checklist is provided as Supporting Information 2: File S1 to ensure transparent reporting of all methodological and analytical aspects. This study employed a multivariable Mendelian randomization (MVMR) design using genetic variation as IVs to assess the causal relationship between BMR and T2D, while excluding the confounding effect of BMI.

### 2.2. Data Sources

Supporting Information 1: Table S2 contains detailed source information for the GWAS data used in this study. Obtained from the IEU Open GWAS database (https://gwas.mrcieu.ac.uk/), the GWAS summary data for BMR in Europeans is derived from the analysis of the UK Biobank [22] phenotypes survey by the Medical Research Council-Integrative Epidemiology Unit (MRC-IEU) consortium [23, 24]. The dataset includes 9,851,867 SNPs from 454,874 participants (https://gwas.mrcieu.ac.uk/datasets/ukb-b-16446/). The Diabetes Meta-analysis of Trans-ethnic Association Studies (DIAMANTE) consortium provided GWAS datasets for T2D (https://kp4cd.org/node/169), with T2D serving as the outcome and encompassing 32 European studies (80,154 cases and 853,816 controls) [25].

In addition, two datasets for BMI, ieu-b-40 (681,275 samples, https://gwas.mrcieu.ac.uk/datasets/ieu-b-40/) and ukb-b-19953 (461,460 samples, https://gwas.mrcieu.ac.uk/datasets/ukb-b-19953/), were obtained from the IEU Open GWAS Project.

### 2.3. IV Selection

Identifying SNPs associated with BMR at the locus-wide significance threshold, a *p* value of 5 × 10^−8^ was chosen to ensure appropriate and potential SNP inclusion. Independent SNP selection, aided by linkage disequilibrium filtering (*r*^2^ = 0.001, window size = 10,000 kb), was conducted as an additional step [26, 27]. Ensuring *F*-statistics were > 10 to minimize weak instrument bias, we calculated SNPs for all included studies, as *F*-statistics represent the magnitude of the genetic instruments. The *F*-statistics were derived using *F* = *R*^2^ (*N* − 2)/(1 − *R*2), where *R*^2^ denoted the proportion of the exposure's variability explained by IVs and *N* represented the sample size of the GWAS for BMR [28]. The final harmonization involved the removal of ambiguous and palindromic SNPs.

### 2.4. Two-Sample MR

Utilizing inverse-variance weighted (IVW), MR–Egger, weighted median (WM), and weighted mode techniques, we investigated the existence of a causal relationship between the genetic risk of exposure and outcome, accounting for the potential of pleiotropy bias. Employing a meta-analysis approach, the IVW method combines Wald estimates of each SNP to provide an overall estimate of the effect of BMR on T2D [29]. Depending on the level of heterogeneity, either a fixed-effects model or a random-effects model was selected [30]. Application of a random-effects IVW model was triggered in cases of significant heterogeneity (*p* < 0.05). To evaluate and ensure the robustness of the MR estimation, a sensitivity analysis was conducted. The multiplicity of the SNPs was evaluated using MR–Egger intercepts, considering a lower multiplicity when the intercept was closer to zero. Heterogeneity was assessed using Cochran's *Q* test. Using the leave-one-out test, we assessed the stability of the MR results by iteratively excluding the IVs one by one to ensure result robustness. The “MR-PRESSO” package in RStudio Version 4.2.1 was utilized for an outlier test, and any potential outliers were subsequently removed. In addition, the *p* values of the associations were corrected for multiple testing using the FDR correction method and were considered statistically significant at *p* < 0.05 [31].

To account for both potential pleiotropy and outliers in an easily interpretable model, we performed Radial MR analyses based on an IVW model with an Egger regression model. The radial regression was used iteratively as the main framework [32]. Outliers were identified based on the contribution of each site to the overall heterogeneity (quantified by Cochran's *Q*, Rucker's *Q* statistic), and SNPs identified as outliers in each of the two sets of Radial MR were eliminated and analyzed again for heterogeneity [33].

### 2.5. Multivariable Mendelian Randomization

To assess the independent association between BMR and T2D and to exclude potential confounding effects of BMI on the results, we performed MVMR analyses [34]. The MVMR approach allows for the simultaneous incorporation of both BMR and BMI into the model as exposure variables, thereby adjusting for the effect of BMI on the association of BMR with T2D at the genetic level. Specifically, we used independent genetic IVs significantly associated with BMR and BMI and performed MVMR analyses based on two-stage least squares (2SLS) [35]. In the first stage, we fitted separate regression models for BMR and BMI on the genetic IVs; in the second stage, both BMR and BMI values predicted in the first stage were included in the regression models to assess the independent causal effect of BMR on T2D.

## 3. Results

### 3.1. SNP Selection and Data Harmonization

Based on the SNP selection criteria, 521 independent variants associated with BMR at genome-wide significance were identified as IVs. Of these, 519 SNPs were available in the T2D summary data (Supporting Information 1: Table S1). Two SNPs (rs3990738 and rs9388498) were missing in the outcome data, and no suitable proxies were identified, leading to their exclusion. After removing palindromic and weak IVs, 505 SNPs remained for analysis.

### 3.2. Influence of the BMR on T2D

**Figure 1:**
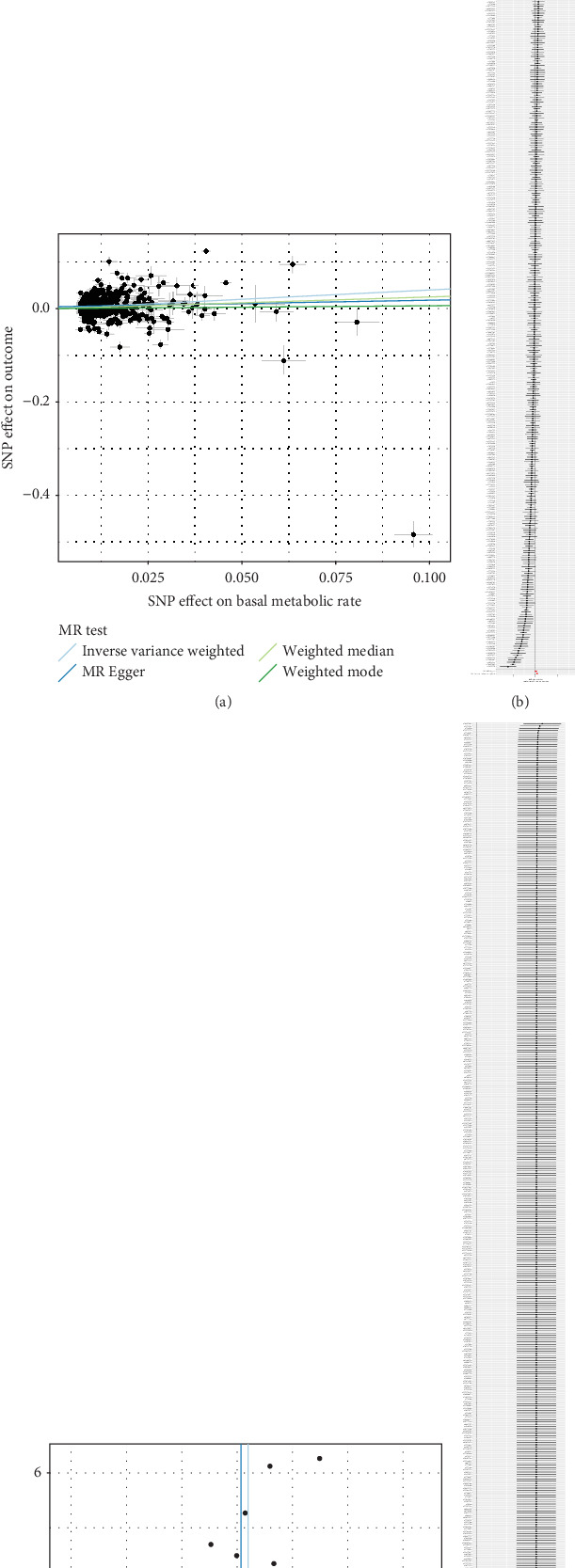
The plots of analysis between BMR and T2D (before eliminating outliers). (a) The scatter plot. (b) The forest plot. (c) The funnel plot. (d) The leave-one-out plot.

In the analysis, the *F*-statistics of the individual SNPs varied from 29.88 to 935.59, with an average value of 81.2. This indicates that there were no weak IVs in the study. The Cochran's *Q* test demonstrated significant heterogeneity (*Q* = 3831.94, *p* < 0.001) (Table 1). To ensure the robustness of the MR analysis results, a multiplicative random-effects model was applied. Utilizing our IVW-MR approach, we found a significant association, indicating that an elevated BMR was linked to an increased risk of T2D (OR, 1.49; 95% CI, 1.31–1.7; *p* < 0.001) (Table 2 and Figure 1a,b); similarly, causality still holds after FPR correction (Table 2). The WM analysis yielded analogous findings, indicating a similar association (OR, 1.28; 95% CI, 1.15–1.43; *p* < 0.001) (Table 2). The MR–Egger analysis did not reveal a statistically significant difference (OR, 1.15; 95% CI, 0.84–1.57; *p* = 0.38), and a similar nonsignificant result was observed with the weighted mode analysis (OR, 1.06; 95% CI, 0.74–1.53; *p* = 0.74) (Table 2).

### 3.3. Sensitivity Analysis

The symmetrical funnel plot indicates minimal heterogeneity in our findings (Figure 1c). Utilizing the leave-one-out approach, a specific SNP was excluded (Figure 1d). According to the MR–Egger regression, the MR pleiotropy test indicated the absence of horizontal pleiotropy (intercept = 0.004, se = 0.002, *p* = 0.07) (Table 1). The MR-PRESSO test confirmed the presence of 91 outliers (Table 3).

**Figure 2:**
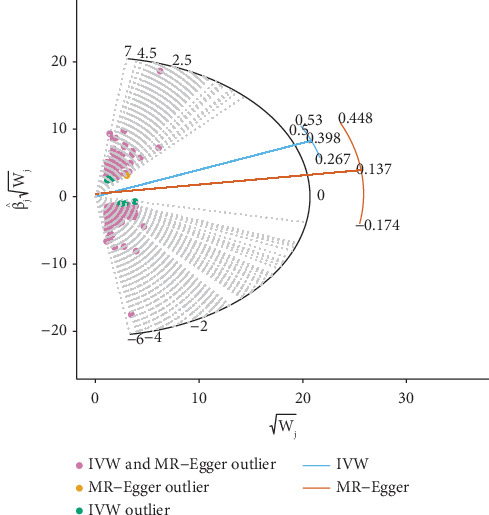
The Radial MR result of BMR on T2D.

**Figure 3:**
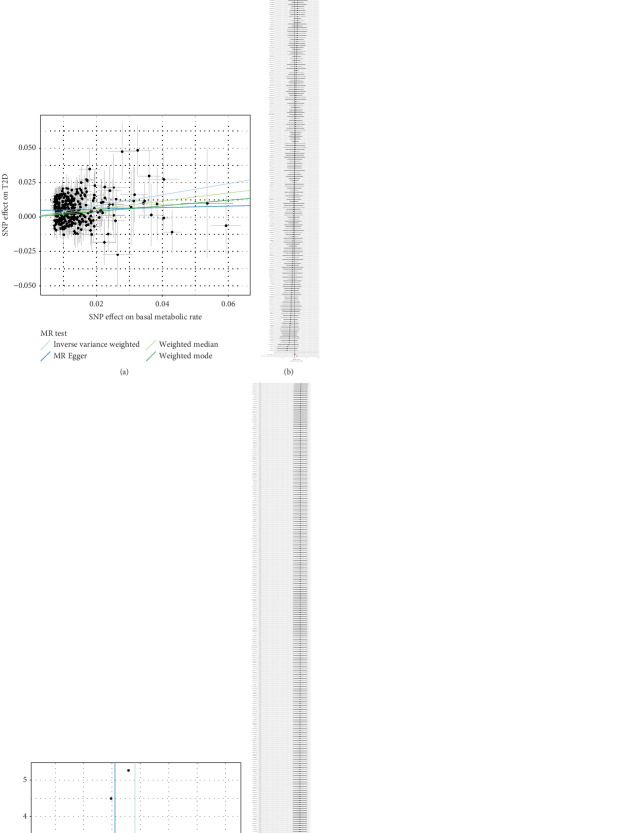
The plots of analysis between BMR and T2D (after eliminating outliers). (a) The scatter plot. (b) The forest plot. (c) The funnel plot. (d) The leave-one-out plot.

To eliminate heterogeneity, outliers were further identified using the Radial MR method. Specifically, 190 outliers were identified (Supporting Information 1: Table S3 and Figure 2), which were removed and analyzed again, and the IVW results remained positive (Supporting Information 1: Table S4 and Figure 3); after removing the outliers, Cochran's *Q* analysis showed no heterogeneity (Supporting Information 1: Table S5), and MR-PRESSO analysis showed no outliers (Supporting Information 1: Table S6).

### 3.4. MVMR Analysis

After performing MVMR analyses to adjust for BMI, we observed that the previous positive results turned insignificant and were observed in two different datasets (Supporting Information 1: Table S7). This change suggests that BMI and body composition may have acted as confounders in the original analyses, leading to biased preliminary results. Specifically, the potential confounding effect of BMI, a factor known to be strongly associated with BMR, was not adequately controlled for in the unadjusted analyses.

## 4. Discussion

Utilizing data from over 500,000 individuals in this two-sample MR study, we consistently identified evidence supporting a causal association between genetically predicted higher BMR and an increased risk of T2D. However, these causal relationships were attenuated after adjusting for BMI. The robustness of the positive causal link was evident in both the primary IVW analysis and several sensitivity analyses addressing directional pleiotropy and heterogeneity. This MR study is, to our knowledge, the first to provide evidence endorsing elevated intrinsic BMR as a causal risk factor for T2D, overcoming challenges related to residual confounding and reverse causation that limited causal inference in prior observational research.

Our findings are consistent with and build upon previous observational studies demonstrating positive associations between directly measured BMR and incident T2D in diverse populations. Studies in Pima Indians [11], Caucasians [12], and Chinese [14] all reported significantly increased risks of future T2D among those with higher BMR at baseline. Despite being suggestive, the observational design of these studies hindered definitive causal conclusions due to potential residual confounding and reverse causation. In this study, we utilized T2D diagnosis status as the outcome rather than quantitative glycemic biomarkers. The binary nature of T2D case status reflects a clinically more relevant endpoint, capturing downstream effects of prolonged glycemic dysregulation. Thus, our findings expand current knowledge by providing evidence at the diagnostic level, indicating a potential causal involvement of increased BMR in T2D pathogenesis.

Several potential mechanisms might elucidate the observed causal relationship between elevated BMR and increased T2D risk. On a cellular level, higher BMR may reflect increased metabolic activity and mitochondrial uncoupling, causing inefficient metabolism and insulin resistance in peripheral tissues [15]. Marked by an increased BMR, the dysregulation of the sympathetic nervous system and hypothalamic–pituitary–adrenal axis can drive diabetogenic effects through processes including gluconeogenesis, lipolysis, and inflammation [16, 36]. Furthermore, individuals with intrinsically higher BMR may compensate through increased food consumption, promoting excessive weight gain and adiposity [37]—major risk factors for T2D. Positive energy balance and obesity over time may be consequences of overeating in response to elevated BMR. In essence, our results genetically support the proposed mechanisms linking higher BMR to subsequent T2D development.

However, the significance of the causal relationship between BMR and T2D disappeared after adjustment for BMI. We suggest that failure to adjust for BMI may have led to confounding effects in the original analyses due to the high correlation between BMR and body weight, body fat content, and lean body mass. After adjusting for BMI by MVMR, the correlations between BMR and some outcome variables were no longer significant, suggesting that there may be important confounding effects of BMI on these associations. The shift from positive to negative results after adjusting for BMI may be due to the high correlation between genetic variation in BMR and BMI, resulting in multivariable analyses in which BMI may have reduced the direct effect of BMR on health outcomes through its pathway of influencing BMR. This suggests the importance of adequately considering relevant factors such as BMI in similar causal inference studies. Although conducting multivariable analyses helped to eliminate the confounding effect of BMI, this change also highlights the complex relationship between BMI and BMR, and future studies may need to isolate these variables more precisely and explore their respective independent effects on health outcomes.

While the use of MR-PRESSO and Radial MR provides a robust mechanism for outlier detection, it is important to note that these methods rely on certain assumptions about the distribution and behavior of the instruments. The identification of outliers could be influenced by various factors, including the presence of weak instruments, horizontal pleiotropy, or even unaccounted confounders that could bias the detection process. In this context, it is crucial to acknowledge that the removal of a large number of outliers could introduce selection bias, particularly if these outliers represent valid genetic variants that are biologically relevant but do not conform to the statistical assumptions of the outlier detection methods. To address this, we conducted sensitivity analyses to assess the impact of outlier removal on results and results remained the same. Future research should refine outlier detection methods to balance robustness with the retention of relevant genetic instruments, ensuring more reliable causal estimates.

Several strengths in our study contribute to the validity of its findings. The large sample size heightened statistical power and precision. BMR was objectively and accurately measured via indirect calorimetry in the GWAS sample. We employed various MR methods under different assumptions to test result robustness. Sensitivity analyses were conducted to scrutinize bias from pleiotropy and heterogeneity. The MR framework minimized confounding and reinforced causal inference from observational data. Nevertheless, certain limitations merit consideration. The reliance on summary-level data constrained the exploration of individual characteristics and subgroups. Generalizability beyond populations of European ancestry may be limited. Despite sensitivity analyses, the complete exclusion of pleiotropic effects of the genetic instruments on T2D through pathways unrelated to BMR cannot be guaranteed. Furthermore, the presence of heterogeneity and horizontal pleiotropy suggests that the observed effects may not be exclusively the result of BMR but may also reflect indirect effects through adiposity, insulin resistance, or physical activity. Moreover, a large number of outliers (190) were identified in this sensitivity analysis, which may have had an impact on the results. In addition, our MR estimate provides an indication of the potential causal influence of genetically increased BMR, and further longitudinal studies are necessary to clarify the temporal dynamics of this relationship. The potential persistence of residual confounding from population stratification is also acknowledged. Of course, MR studies only provide causal inferences and further follow-up studies are needed to confirm the underlying mechanisms.

## 5. Conclusions

In summary, our MR study provides novel evidence supporting the possibility that a genetically determined higher BMR may be associated with T2D risk. This improves etiological understanding of intrinsic metabolic factors in T2D pathogenesis beyond conventional risk factors like obesity. Our findings suggest that interventions targeting cellular metabolic homeostasis and energetics could potentially have benefits for T2D prevention. This merits additional investigation in clinical and translational research. Further studies are needed to confirm our results in diverse populations, elucidate underlying biological mechanisms, and explore clinical and public health implications of the observed causal relationship between elevated BMR and increased T2D risk.

## Figures and Tables

**Table 1:** Results of heterogeneity test and pleiotropy test of instrumental variables.

**Outcome**	**Exposure**	**Heterogeneity**		**Pleiotropy**	
		**Q** ** statistic (IVW)**	**p** ** value**	**MR–Egger intercept**	**p** ** value**
Type 2 diabetes (DM)	Basal metabolic rate (BMR)	3831.94	0	0.004	0.07

**Table 2:** Association between basal metabolic rate and risk of Type 2 diabetes.

**Outcome**	**Exposure**	**N** ** SNPs**	**Methods**	**OR (95% CI)**	**p**	**FDR adjusted ** **p**
Type 2 diabetes (DM)	Basal metabolic rate (BMR)	505	Inverse-variance weighted	1.49 (1.31–1.7)	*p* < 0.01	*p* < 0.01
			MR–Egger	1.15 (0.84–1.57)	0.38	0.51
			Weighted median	1.28 (1.15–1.43)	*p* < 0.01	*p* < 0.01
			Weighted mode	1.06 (0.74–1.53)	0.74	0.75

**Table 3:** MR-PRESSO test results.

**Outcome**	**Exposure**	**Raw**			**Outlier corrected**			**Global ** **p**	**Number of outliers**	**Distortion ** **p**
		**OR**	**95% CI**	**p**	**OR**	**95% CI**	**p**			
Type 2 diabetes (DM)	Basal metabolic rate (BMR)	1.49	1.32–1.70	1.6E − 09	1.48	1.35–1.61	1.56E − 16	< 0.001	91	0.723

## Data Availability

All data generated or analyzed during this study are included in this published article.

## Supporting Information

Supporting Information 1 Table S1. The results of SNP selection and data harmonization. Table S2. Detailed information for the GWAS data. Table S3. The outliers identified by Radial MR. Table S4. Association between basal metabolic rate and risk of Type 2 diabetes (after eliminating outliers). Table S5. Results of heterogeneity test and pleiotropy test of instrumental variables (after eliminating outliers). Table S6. MR-PRESSO test results (after eliminating outliers). Table S7. Results of MVMR.

Supporting Information 2 File S1. STROBE-MR checklist.
